# The Tenth Annual Meeting of the Missouri Veterinary Medical Association

**Published:** 1901-12

**Authors:** B. F. Kaupp

**Affiliations:** Secretary


### SOCIETY PROCEEDINGS.

### THE TENTH ANNUAL MEETING OF THE MISSOURI VETERINARY MEDICAL ASSOCIATION.

The tenth annual meeting of this State Association was called to order in the lecture-rooms of the Kansas City Veterinary College at 10 A.M., October 22, 1901, by the President, Dr. F. W. O'Brien, of Hannibal. The following veterinarians were present: Drs. M. McNally, F. W. O'Brien, B. F. Kaupp, W. E. Martin, J. W. Connaway, Missouri State Experiment Station; Stanley Smith, R. C. Moore, Charles Doerrie, S. Stewart, R. H. Carswell, L. D. Brown, F. F. Brown, Walter Warren, W. A. Nixon, H. V. Patterson, E. Brainerd, A. G. G. Richardson, E. M. Nighbert, E. Lee, A. D. Knowles, W. A. Thomas, J. S. Grove, J. D. Sprague, J. S. Anderson, J. H. Gain, John Nott, Frank Linscott, Charles Saunders, C. H. Davies, W. C. Barth, A. Trickett, W. F. Lavery, H. H. George, N. B. Smith, W. R. Cooper, C. A. Clawson, W. R. Pick, A. N. Reber, W. N. Hobbs, W. R. Andress, A. W. Swedburg, A. Plummer, Fourth United States Cavalry; A. T. Peters, Nebraska Experiment Station; A. MacDonald, Fourth United States Cavalry, and others. There were many visitors who were not veterinarians, but interested in the profession; also about 100 veterinary students. The minutes of the previous meeting were read, after which a report of the Legislative Committee, by Dr. D. F. Lucky, member of said committee, was read. Mr. J. A. McLane, a representative from Jackson County, gave a good report of the efforts to secure needed State veterinary legislation at the last session of the State Legislature.

Moved by Dr. J. W. Connaway, seconded and carried, that a committee be appointed by President O'Brien to draft resolutions commending the Legislative Committee for its work. The committee was Drs. Connaway (Chairman), S. Smith, and L. D. Brown. The Legislative Committee's report was accepted and the meeting adjourned to luncheon, to meet again at 2 P.M.

At 2 P.M. the meeting was again called to order by the President, when the following papers were presented: "An Enzootic Attack of Chorea Among Cattle," by Dr. A. D. Knowles; "Sclerastoma Tetracanthus of the Horse," by Dr. W. E. Martin—the doctor gave a good report of some cases, and illustrated his paper by demonstrations of the parasite and a portion of the affected bowel; "Pleurisy of the Horse," by Dr. F. F. Brown; "Acute Rheumatism of the Horse," by Dr. F. J. Menestrina.

Under reports of cases Drs. L. D. Brown and F. W. O'Brien gave some interesting cases of parturient paresis of the cow, using Schmidt's treatment, which was discussed by Drs. Nighbert, Knowles, Stewart and others. Dr. F. F. Brown was elected a member of the Association. The following officers were then elected for the ensuing year: President, Dr. J. W. Connaway, Columbia, Mo.; Vice-President, Dr. Charles Doerrie, Booneville, Mo.; Secretary-Treasurer, Dr. B. F. Kaupp, Kansas City, Mo. A motion was then made, seconded, and carried to adjourn, to meet at 7.30 P.M.

At 7.30 P.M. the meeting was called to order by the President, when the following programme was presented: "Immunizing Northern-bred Cattle Against the Southern Cattle Fever," by Dr. J. W. Connaway, of the Missouri State Experiment Station—the doctor gave a very interesting and instructive address, using charts, pictures, and instruments to illustrate the present methods used at the Missouri State Experiment Station of immunizing our Northern cattle against the Southern cattle fever; "Tuberculosis of Cattle and Swine," by Dr. W. R. Cooper; "Actinomycosis of Cattle," by Dr. H. H. George. These papers brought out a good discussion, which was enjoyed by all present. Dr. A. Plummer, Fourth United States Cavalry, recently of the Philippine Islands, gave some interesting accounts of the filaria oculi in the horse on the islands. He said that it had been noted that the affection occurred in horses in the low, marshy part of the country, but not noticed on the highlands. The operation of paracentesis always resulted in recovery. Operation was performed on inferior parts of the cornea, allowing the worm to escape with the aqueous humor. After-treatment consisted of cold applications and to keep the eye darkened.

It was moved by Dr. Connaway, seconded and carried, that we invite the American Veterinary Medical Association to meet in Kansas City in 1902. Moved by Dr. Stewart, seconded and carried, that the exact date and place of next meeting be left with the Executive Committee. The meeting then adjourned until 8 A.M., October 23d.

At 8 o'clock, October 23, 1901, a surgical and dental clinic was held, which was joined in by the twenty-ninth regular meeting of the Missouri Valley Veterinary Association. Dr. E. M. Nighbert demonstrated the excellences of the new operating-table advertised. Dr. J. S. Anderson, of Seward, Neb., demonstrated the operation of hyovertebrotomy for the cure of cribbing; also the operation of arytenoiderrhaphy for the cure of "roaring." Drs. W. E. Martin and F. W. O'Brien demonstrated the passage of the stomach-tube for gastric tympany, also irrigating and flushing the stomach. The procedure met with marked success, and was highly commended by those present. After luncheon the veterinarians visited the plant of the Armour Packing Company, where a fine display of pathological specimens had been collected and arranged under the direction of Drs. A. G. G. Richardson and W. R. Cooper and other veterinarians of the United States Meat Inspection force at this point. This exhibit consisted of pathological lesions found in cattle, calves, sheep, and swine. Among the specimens of special interest were noted tuberculosis of cattle and swine, showing lesions in the carcass (in glands, serous membranes, and bones), lungs, liver, spleen, etc.; actinomycosis of cattle, lesions in the head and lungs; cirrhosis, echinococcus, and abscess of the liver; distomatosis lesions in liver and lungs of cattle; caseous lymph adenitis and nodular disease of sheep; hog cholera in carcass and viscera, and many other interesting specimens.

At 3 P.M. the party was conducted by the local veterinarians to the cattle show and sales, where an opportunity was afforded to make a study of judging cattle, and hundreds of fine specimens of the best beef breeds, including the Hereford, Shorthorn, and Poll Angus, were seen.

At 8 P.M. the local veterinarians entertained about forty visiting veterinarians at the Kansas City Horse Show, which was held in the famous Kansas City Convention Hall, where all had an enjoyable time, after which the party disbanded, ending one of the most successful meetings in the history of the Association.

B. F. Kaupp, D. V. S.,
*Secretary.*

